# Use of the ShangRing circumcision device in boys below 18 years old in Kenya: results from a pilot study

**DOI:** 10.7448/IAS.20.1.21588

**Published:** 2017-07-12

**Authors:** Quentin D. Awori, Richard K. Lee, Phillip S. Li, Jared N. Moguche, Dan Ouma, Betsy Sambai, Marc Goldstein, Mark A. Barone

**Affiliations:** ^a^ Clinical Research Department, EngenderHealth, Nairobi, Kenya; ^b^ Center for Male Reproductive Medicine and Microsurgery, Department of Urology, Weill Cornell Medical College, New York, NY, USA; ^c^ Biostatistics Department, Bon Sante Consulting, Nairobi, Kenya; ^d^ Clinical Research Department, EngenderHealth, New York, NY, USA

**Keywords:** Male circumcision, ShangRing, circumcision device, adolescents, infants

## Abstract

**Introduction**: Male circumcision is a proven prevention strategy against the spread of HIV. The World Health Organization’s new 2016–2021 strategic framework on voluntary medical male circumcision (VMMC) targets 90% of males aged 10–29 years to receive circumcision by 2021 in 14 priority sub-Saharan countries while anticipating an increase in the demand for infant circumcision. It also states that the use of circumcision devices is a safe and efficient innovation to accelerate attainment of these goals. The primary objective of this pilot study was to evaluate the safety and acceptability of the ShangRing, a novel circumcision device, in boys below 18 years of age.

**Methods**: A total of 80 boys, 3 months to 17 years old, were circumcised using the no-flip ShangRing technique. All rings were removed 5–7 days later. Participants were evaluated weekly until the wound was completely healed. Data on procedure times, adverse events (AEs), time to clinical wound healing and satisfaction were recorded and analysed.

**Results**: Nearly all (79/80, 98.8%) circumcisions were successfully completed using the no-flip ShangRing technique without complications. In one (1.2%) case, the outer ring slipped off after the foreskin was removed and the procedure was completed by stitching. The mean circumcision and ring removal times were 7.4 ± 3.2 and 4.4 ± 4.2 min, respectively. There were four (5%) moderate AEs, which were managed conservatively. No severe AEs occurred. The mean time to complete clinical healing was 29.8 ± 7.3 days. Participants or their parents liked ShangRing circumcision because it improved hygiene, was quick and possessed an excellent cosmetic appearance. Most (72/80, 94.7%) were very satisfied with the appearance of the circumcised penis, and all (100%) said they would recommend circumcision to others.

**Conclusions**: Our results suggest that no-flip ShangRing VMMC is safe and acceptable in boys below 18 years of age. Our results are to be compared those seen following ShangRing VMMC in African men. Further study with larger sample sizes are needed to explore the scalability of the ShangRing in larger paediatric cohorts in Africa. We believe that the ShangRing has great potential for use in all age groups from neonates to adults, which would simplify device implementation.

## Introduction

Male circumcision is one of the oldest surgical procedures. Its efficacy in reducing the spread of HIV was demonstrated in three randomized controlled trials (RCTs) conducted in Kenya, Uganda and South Africa. Circumcised men are 60% less likely to acquire HIV heterosexually compared to uncircumcised men [1–3]. This protective effect has been shown to endure for at least five to six years [4,5]. Based on the findings from the RCTs and recommendations from the World Health Organization (WHO) and the Joint United Nations Programme on HIV/AIDS (UNAIDS), 14 sub-Saharan African countries, including Kenya, have been implementing voluntary medical male circumcision (VMMC) as part of their national HIV prevention programmes [6,7]. The original goal to provide VMMC to 80% of adult and newborn males by 2016 was not achieved [8,9].

Boys under 18 years old and in particular those in the 10–14 and 15–17 age groups are important strategically for long-term sustainability of VMMC programmes in Africa [8]. In fact, the WHO VMMC 2016–2021 strategic framework specifically aims to have 90% of males between 10 and 29 years of age circumcised by the year 2021 [10]. Kenya has prioritized infants 0–60 days of age and adolescents 10–14 years, while still including males 15 years and above in its 2014–2019 VMMC strategic plan [11]. As communities embrace VMMC, the demand from parents of older boys has increased. In some communities in Tanzania, a preference for prepubertal circumcision, that is before 12 years, as opposed to post-pubertal, and a preference for medical as opposed to traditional circumcision have been reported [12].

Service delivery challenges that may impede the implementation of VMMC especially in public facilities include staff shortages and work overload, particularly when there are no dedicated VMMC staff and those from other departments have to also provide VMMC. Additionally, the relative technical difficulty of the recommended conventional circumcision techniques limits the number of clients who can receive services at any time [13]. The use of circumcision devices could facilitate scale-up of VMMC as device-assisted circumcisions take much less time, i.e. more circumcisions can be performed within an available time window. Comparative studies have shown a higher preference for device-assisted vis a vie conventional circumcision among VMMC clients and male circumcision providers (MCPs) [14,15]. Additionally, the procedures could be safely performed by non-physician clinicians who are more widely available [16,17]. To achieve the target of 90% VMMC coverage in 10–29-year olds in sub-Saharan Africa, 5 million circumcisions will need to be done annually through 2021 [10].

While there are many circumcision devices currently in use or under study, none has yet received WHO pre-qualification for use across all ages. The ShangRing is a novel male circumcision device invented in China in 2005. In 2015, it obtained WHO pre-qualification for use in men and boys 13 years old and above using the original ShangRing technique [18]. The original ShangRing technique has been extensively studied in African adults [14–16,19] and in adolescents aged 13 years and above [20].

In the original ShangRing technique, the inner ring is first placed around the glans penis, outside the foreskin; the foreskin is then everted over the inner ring. The outer ring is then secured over the inner ring with the foreskin sandwiched between the rings [21].

A notable variation to the original ShangRing technique is the no-flip technique. It was first described by Yan et al. [22] in 2008 in a case series study that enrolled 824 boys 4–15 years old for treatment of phimosis and redundant prepuce. In the no-flip technique, the inner ring is placed inside the foreskin, around the glans penis. The outer ring is then clamped around the inner ring, on the outside of the foreskin, sandwiching the foreskin between the rings; there is no need to flip the foreskin over the inner ring, hence the name no-flip. Yan reported a mean ShangRing placement (with foreskin removal) time of 2.6 ± 1.6 min, four cases of infection (0.6%) and 21 of oedema (3.2%); all resolved conservatively [22].

In a three-armed RCT conducted in China among children 4–11 years of age, the two ShangRing techniques and conventional circumcision were compared [23]. While the original ShangRing arm underwent ring removal seven days post-placement, the no-flip arm underwent removal after 21 days if spontaneous detachment of the ring had not occurred. Both ShangRing techniques had significantly shorter circumcision times and less bleeding compared to the conventional technique. There was no significant difference in adverse event (AE) rates between the groups. About 75% of the boys in the no-flip group experienced spontaneous detachment. The authors of both studies concluded that the no-flip method may be better than the original ShangRing technique in children [23].

A meta-analysis in China compared the original ShangRing technique to the no-flip technique and has found the latter to have fewer complications, lower incidence of postoperative oedema and mild postoperative pain [24]. We are not aware of any reported use of the no-flip technique outside China.

The primary objective was to evaluate the safety and acceptability of the no-flip ShangRing technique in boys below 18 years in Kenya. Secondary objectives included (1) evaluating the device placement and removal times and (2) evaluating the course and duration of healing of the wound following circumcision.

## Methods

### Study design and setting

This was a pilot study of the ShangRing for VMMC in 80 boys conducted in Homa Bay, Kenya. The study site was the VMMC clinic at the Homa Bay County Referral Hospital. Recruitment and follow-up took place from July through November 2013.

### Study participants

Participants were recruited from clients seeking VMMC. To be eligible, participants had to be below 18 years of age; accompanied by a parent, guardian or legally acceptable representative (hereafter parent), in good health, free of sexually transmitted infections, 2.5 kg or more, and with a penile shaft over 1 cm in length. The parent was required to have a good understanding of the study, agree to bring the participant for follow-up and have a cell phone or access to one. Participants were excluded if they had a known allergy to lidocaine, bleeding disorder, active genital infection or genitourinary abnormality that contraindicated elective surgery under local anaesthesia or circumcision.

### Procedures

Participants or their parents were interviewed to gather baseline demographics. Participants were examined clinically to verify eligibility. One doctor and two nurses, all trained and experienced in conventional surgical and the original ShangRing technique, performed the ShangRing circumcisions. Previous to commencing the study, only the doctor had received training in the no-flip technique in China. During the study, the two nurses learned the no-flip modification and were proficient after having done five placements.

Participants were administered paracetamol 30 minutes prior to circumcision. A dorsal penile nerve and ring block was administered using 1% lidocaine.

After device placement and foreskin removal, the parent was counselled on postoperative care. Participants were present and included in the discussion if they were older and appeared to understand. Device removal took place 5–7 days after device placement. Follow-up visits were scheduled on 14, 21, 28, 35 and 42 days post-placement. Parents/participants were encouraged to return at any time if there was complication, excessive discomfort or other problems. At each visit, a genital examination and interview were conducted. Photographs were taken to document clinical wound healing and AEs. Participation ended on the visit that a study clinician determined the circumcision wound was clinically healed. A final interview was conducted during this visit or at 42 days in those participants whose wounds had not yet healed.

Most data were collected on mobile devices at each study visit using Open Data Kit (ODK®), an open-source mobile application for data collection [25].

### Outcomes

AEs were classified based on the definitions in the Population Services International (PSI)/WHO Adverse Event Action Guide for Male Circumcision [26]. We modified the definition of wound dehiscence to account for the fact that no sutures are used with the ShangRing and that healing is by secondary intention [14]. AEs were categorized as mild, moderate or severe. A mild AE was any AE that though noticeable or reported by the participant, was short lived and required no treatment. Events of mild severity were considered to be within the normal range of circumcision sequelae. Moderate AEs would interfere with normal activity and comfort, but responded to simple measures, while not affecting the general course of healing. Severe AEs would interfere significantly with the participant’s normal activity or comfort and needed advanced therapeutic measures in their management. AEs that have been seen after ShangRing VMMC include pain, bleeding, oedema, infection and wound disruption. The detailed AE listing used during the study can be found in the supplemental material for the manuscript. The rates of moderate and severe AEs were used to assess safety of the no-flip ShangRing procedure. MCPs were trained and experienced in assessing AEs from previous ShangRing studies [14,16,19,27].

During the no-flip procedure, we noted the anaesthesia time and time for device placement including foreskin removal. The former span from when injectable anaesthesia was administered to when it took effect, while the latter span from when the foreskin was held using forceps for insertion of the inner ring to when removal of the foreskin was complete. The duration of device removal was recorded starting from when the outer ring was opened until when the inner ring was removed. Ease of using the ShangRing was determined by the number of reported difficulties during the placement and removal procedures.

The length of time for clinical wound healing was reported from the day of device placement to the follow-up visit when the participant was determined to be healed. Clinical healing was defined as complete re-epithelization and keratinization of the wound and was subjectively assessed during the clinical examination by the MCP.

Prior to ring placement, anaesthesia was always confirmed. However, we did not measure pain during (if any) or after ring placement. We evaluated pain during ring removal. Among participants above 7 years of age, a visual analogue scale (VAS) which ranged from 0-10 was used [28]. Reported scores of 0–2, 3–6 and 7–10 were recorded as mild, moderate and severe, respectively. The Children’s Hospital of Eastern Ontario Pain Scale (CHEOPS) was used in those participants 7 years old and below as it is better suited in younger children [29]. On this scale, which ranges from 4 to 13, we categorized scores of 4–6, 7–10 and 11–13 to be mild, moderate and severe, respectively.

To evaluate satisfaction and acceptability, boys and their parents were interviewed during the last visit. They were asked open-ended questions related to what they liked and disliked about the circumcision, with the responses grouped into categories by the investigators after the study ended. They were also asked about their level of satisfaction (very satisfied, somewhat satisfied, somewhat dissatisfied and not satisfied at all) with the appearance of the circumcised penis and whether they would recommend circumcision to another boy.

### Statistical analysis

Means, standard deviations and percentages were used to describe quantitative variables. To compare time to anaesthetic effectiveness, durations of device placement and removal procedures and clinical wound healing between the different age categories, the largest group was used as the reference. Student *t*-test was used to determine the level of significance; alpha level was set to 5%. No missing data were imputed.

### Ethical approval

Ethical approval was obtained from the Weill Cornell Medical College and the Kenya Medical Research Institute. Regulatory approval was received from the Kenya Pharmacy and Poisons Board. The study was registered on ClinicalTrials.gov, identifier: NCT01891409. Informed consent was obtained from the boy’s parent on the day of recruitment. Assent was taken from participants above the age of seven years who understood the study procedures.

## Results

All 80 participants who had been taken through informed consent and screened were found to be eligible and were recruited into the study. Baseline socio-demographic characteristics are shown in Table 1. More than half (44/80, 55.0%) were in the 1-9 year age group had 44/80. The most frequent presenting parent was the mother (51/80, 63.7%). Nearly all (78/80, 97.5%) participants were of Luo ethnicity. The most commonly reported primary reason for seeking VMMC was hygiene in 48 (60%) participants followed by protection against HIV infection in 28 (35%) participants.

**Table 1.** Sociodemographic characteristics of study participants (*n* = 80)

Characteristics	*n*	(%)
Age group (years)		
<1	6	(7.5)
1–9	44	(55.0)
10–12	10	(12.5)
13–14	12	(15.0)
15–17	8	(10.0)
Presenting parent		
Mother	51	(63.7)
Father	15	(18.8)
Guardian	10	(12.5)
Sibling	2	(2.5)
Other	2	(2.5)
Primary reason for seeking VMMC		
Hygiene	48	(60.0)
HIV protection	28	(35.0)
Social/religious reason	3	(3.7)
Medical therapy	1	(1.3)
Level of education		
Not in school (too young)	21	(26.3)
Early chid development/pre-primary	19	(23.7)
Lower primary	7	(8.8)
Upper primary	30	(37.5)
Secondary	3	(3.7)

Almost half (38/80, 47.5%) of the participants had penile adhesions. Of these, 68.4% (26/38) were mild, 21.0% (8/38) were moderate and 10.5% (4/38) were severe. The mean age of participants who had penile adhesions was just under half that of participants without penile adhesions (4.1 ± 4.1 and 8.8 ± 4.2, respectively). All were successfully released prior to device placement. In 11.3% (9/80) of the boys, a dorsal slit was required in order to insert the inner ring.

Overall, the mean time taken for the injectable anaesthesia to take effect was 4.7 ± 3.5 min, while placement of the ShangRing device (with removal of the foreskin) took 7.4 ± 3.2 min. None of the age-specific findings when compared to the reference were significant (Table 2).

**Table 2.** Time for anaesthesia to take affect and for completion of ShangRing placement and removal procedures (*n* = 80)

Time	Age	*n*	Minutes (SD)	*p*
Anaesthesia to take affect	<1 year	6	4.3 (2.6)	0.62
	1–9 years	44	4.7 (3.8)	Ref
	10–12 years	10	4.8 (3.3)	0.47
	13–14 years	12	4.9 (3.6)	0.43
	15–17 years	8	4.6 (3.2)	0.53
	**All**	**80**	**4.7(3.5)**	
ShangRing placement and foreskin removal	<1 year	6	10.3 (4.9)	0.09
	1–9 years	44	7.4 (2.9)	Ref
	10–12 years	10	8.9 (2.6)	0.07
	13–14 years	12	7.1 (4.0)	0.59
	15–17 years	8	6.1 (4.5)	0.77
	**All**	**80**	**7.2(3.2)**	
ShangRing removal	<1 year	6	1.5 (0.8)	0.99
	1–9 years	44	3.7 (2.5)	Ref
	10–12 years	10	4.7 (1.7)	0.07
	13–14 years	12	4.8 (2.2)	0.07
	15–17 years	8	4.3 (1.7)	0.20
	**All**	**80**	**4.4(4.2)**	

Nearly all (79/80, 98.8%) ShangRing placements/removals were successfully completed without difficulties (Table 3). In one (1.2%) participant aged 9, the outer ring slipped off immediately after the ShangRing had been applied and the foreskin removed. The circumcision wound was closed using sutures as in conventional circumcision, while the client did not otherwise experience any untoward effect.

**Table 3.** Surgical difficulties

Surgical difficulties	*n*	(%)
ShangRing placement and foreskin removal (n = 80)		
Outer ring slipped off immediately postoperatively^a^	1	(1.3)
None	79	(98.7)
ShangRing removal (n = 79)		
Tight scab^a^	5	(6.3)
None	74	(93.7)

^a^All managed without any sequelae.

All participants attended their removal visit and had the ShangRing removed. Mean ring removal time was 4.4 ± 4.2 min; none of the differences between the age groups when compared to the reference was significant (Table 2). In 5 (6.3%) cases, ring removal was reported to be difficult due to a tight scab around the inner ring (Table 3).

There were no severe AEs and four (5%) moderate AEs, all of which were definitely related to the procedure (Table 4). A summary of the AEs and how they AEs were managed is shown in Table 4. All AEs were treated conservatively and resolved without further incident. Wound disruption was the most common AE and was managed conservatively with alternate day change of dressing.

**Table 4.** Adverse events and management

Age	AE	Occurrence post-placement	Management
10 years	Pain during removal	5 days	Paracetamol 500 mg as a STAT dose
5 years	Wound disruption^a^	6 days	Wound moistened and ring removed following day
12 years	Wound disruption	13 days	Dry scab removed from wound
15 years	Wound disruption with mild infection	9 days	Wound cleaned, dressing applied and antibiotics administered

^a^AE experienced while ShangRing was still in place.

The mean pain experienced during ShangRing removal by participants 7 years old and below (*n* = 37), as measured using the CHEOPS scale (4 = no pain to 13 = worst possible pain), was 8.8 ± 1.8. In participants above 7 years of age (*n* = 34), the mean pain reported at removal was 4.1 ± 1.6 using the VAS (0 = no pain to 10 = worst possible pain). Pain scores were missing for nine participants.

Loss to follow-up was minimal (4/80, 5.0%). Of the 76 participants who completed the study, the majority (74/76, 97.4%) were healed before or at the last planned follow-up which was 42 days post-placement. The remaining two participants (2.6%), who were not healed at 42 days, did not return for additional visits as requested. Overall, mean time to complete clinical healing was 29.8 ± 7.3 days (Table 5). The percentage of participants determined to be clinically healed at each follow-up visit were as follows: 0% (0/74) on day 7, 2.7% (2/74) on day 14, 21.6% (16/74) on day 21, 40.5% (30/74) on day 28, 24.3% (18/74) on day 35 and 10.8% (8/74) on day 42.

**Table 5.** **Time to clinical healing (days) by age group (*n* = 74)**^a^

Age group	*n*	Mean	(SD)	*p*
<1 years	6	19.7	(2.8)	0.99
1–9 years	44	29.9	(7.9)	Ref.
10–12 years	10	32.0	(4.5)	0.13
13–14 years	9	31.4	(5.0)	0.23
15–17 years	5	35.5	(0.0)	0.99
**All**	**74**	**29.3**	**(7.3)**	

^a^Healing not documented in two participants and data missing from four participants lost to follow-up.

Preferences and satisfaction of clients are shown in Table 6. Participants and/or their parents reported that they liked the ShangRing circumcision because it improved hygiene, was quick or left a nice cosmetic appearance upon healing. Nearly three-quarters (51/76, 71%) reported there was nothing they disliked about the procedure, while some reported that they (or their child) experienced more pain than expected or indicated that the healing took longer than anticipated. Most (72/76, 94.7%) participants and/or parents said that they were very satisfied with the appearance of the circumcised penis. Additionally, all (100%) participants and/or parents said that they would recommend ShangRing circumcision to another parent or friend of the same age as them.

**Table 6.** Satisfaction with the ShangRing among participants

Interview question	*N* = 76	(%)
Things liked about circumcision^a^		
Improved hygiene	74	(97.4)
Cosmetic appearance	57	(75.0)
Circumcision took a short time	51	(67.1)
Things disliked about circumcision^a^		
None	54	(71.1)
More pain than expected	12	(15.8)
Circumcision wound took long to heal	5	(6.6)
Wound care during healing was difficult	4	(5.3)
Not the best cosmetic appearance	1	(1.3)
Satisfaction with appearance		
Very satisfied	72	(94.7)
Somewhat satisfied	4	(5.3)
Somewhat dissatisfied	0	(0)
Not satisfied at all	0	(0)
Would recommend the ShangRing to another friend/parent		
Yes	76	(100)
No	0	(0)

^a^Multiple responses possible.

## Discussion

This study is the first to evaluate the safety and acceptability of ShangRing circumcision in children below 13 years of age in Africa. It is also the first to evaluate the no-flip technique in Africa in any age group. Our findings suggest that the ShangRing is safe and acceptable for use in boys. This corroborates results from studies in adolescents and adults in Africa using the original ShangRing technique, including placement and removal times [14–16,20]. Should further research confirm our results, a unique advantage of the ShangRing would be that it can be used safely in all ages from neonates to adults. This would facilitate VMMC scale-up while simplifying service delivery, training, supervision and supply chain management.

Neither phimosis nor preputial adhesions prevented successful ShangRing placement. This has also been seen in previous studies; in fact, Yan et al. and Pan et al. used the ShangRing to treat phimosis [22,23]. No mention is made of any exclusions due to penile adhesions. To allow for insertion of the inner ring, adhesions were released and/or a small dorsal slit was made in the foreskin as necessary, while participants were under local anaesthesia. With the PrePex circumcision device, 34.2% of 13–17-year olds were ineligible for circumcision due to phimosis or preputial adhesions, with more ineligibles at younger ages (13.3% of 17 year olds vs. 51.9% of 13 year olds) [30].

We report a moderate AE rate of 5% (4/80), with no severe AEs. These results are also similar to those with the no-flip technique in children in China [23,31]. In a study of ShangRing circumcisions in Ugandan adolescents 13–17 years old, the moderate AE rate was 1.3% (4/334) [20]. Wound disruption was the most common AE we observed, similar to ShangRing circumcision in adults in Kenya and Zambia [14]. Unlike wound disruption following conventional circumcision, usually associated with infection, infection has rarely been reported following ShangRing circumcision [14,16].

We report one device placement failure; the outer ring slipped off immediately after ShangRing device placement and foreskin removal was completed. In the study of Ugandan adolescents [20], three (0.9%) similar cases were reported. In all, the circumcisions were completed using sutures as in conventional techniques. Such problems with device placement have been reported neither in boys or men in China nor in the over 1500 ShangRing placements in adults in Kenya and Zambia [14,32]. We recommend that that providers using the ShangRing should also be competent in conventional circumcision technique should a complication arise..

We saw an apparent increase in clinical healing times with increasing age, but this was not consistent with the oldest group nor statistically significant. Generally though, healing following circumcision has been found to be quicker in younger boys possibly due to lower levels of pro-inflammatory cytokines released in the healing wound [33].

Given that circumcision of newborns and younger boys is less likely to lead to complications, heal faster than in adults and is cost-effective [34,35], future VMMC programmes may find it advantageous to prioritize these age groups. Early infant male circumcision (EIMC) is defined as circumcision below the age of 60 days. Lower AE rates have been seen following circumcision in early infants compared to older boys [35]. Nonetheless, the current WHO approved EIMC devices suffer from some limitations. For example, the Mogen and Gomco clamps routinely require closure with sutures in boys over 60 days old [34]. The Plastibell is appropriate only in situations where follow-up is reliable as serious complications can occur if parts of the device are retained [36].

Challenges in accessing healthcare services and prevailing cultural attitudes may preclude many parents from seeking VMMC for their infants before they are 60 days old. Some parents of newborns may also want to defer the circumcision of their children to a later age [37]. Six of the participants in our study were aged 3–11 months. If our results are confirmed in larger numbers of infants and boys, the ShangRing could be an alternative for infants older than 60 days and for boys whose follow-up is not certain. In the latter case, spontaneous detachment of the ShangRing would likely occur as has been observed to safely take place in adults and children when the ring was left on for longer than seven days [19,23]. This would also alleviate the need for a removal-day visit, reducing the burden of services at the health facilities.

The ShangRing technique was found to be highly acceptable to boys and their parents, similar to previous reports in adults and adolescents [14,16,20]. However, as multiple responses to our interview question were possible, it is not clear if participants favoured ShangRing circumcision for reasons more specific to the device (e.g. short circumcision procedure time and a better resulting cosmesis) rather than reasons related more generally to circumcision (e.g. improved hygiene). Most boys and their parents said there was nothing that they disliked about the circumcision, while all participants were either very or somewhat satisfied with how the wound healed. High acceptability of the device could subsequently increase the demand for VMMC.

Limitations of the study include the small sample size and uneven age distribution of participants. Only six boys were below 1 year of age, and hence findings from this group, though positive, are difficult to generalize. Further, pain experienced during administration of anaesthesia and after ring placement was not evaluated. Time to healing could have been overestimated because the follow-up visits were at seven-day intervals. As this was a case series, the performance of the no-flip ShangRing  technique compared to other devices or the original ShangRing technique was not possible, as would have been the case in an RCT.

## Conclusions

Our results suggest that the no-flip ShangRing technique is safe and acceptable for VMMC in boys below 18 years of age in Africa. Given that the ShangRing has received WHO pre-qualification for use in boys and men 13 years of age and older [18], should further studies confirm our results in larger paediatric cohorts in Africa, the ability to use the ShangRing in all ages from adults to neonates would simplifying VMMC implementation.
